# Construction of transgene-amplified CHO cell lines by cell cycle checkpoint engineering

**DOI:** 10.1186/1753-6561-7-S6-O7

**Published:** 2013-12-04

**Authors:** Kyoung Ho Lee, Kohsuke Honda, Hisao Ohtake, Takeshi Omasa

**Affiliations:** 1 Department of Biotechnology, Graduate School of Engineering, Osaka University, 2-1 Yamadaoka, Suita, Osaka 565-0871, Japan; 2 Institute of Technology and Science, The University of Tokushima, 2-1 Minamijosanjima-cho, Tokushima 770-8506, Japan

## Introduction

Dihydrofolate reductase (DHFR)-mediated gene amplification has been widely used to establish high-producing mammalian cell lines [1-3]. However, since gene amplification is an infrequent event, in that many rounds of methotrexate (MTX) selection to amplify the transgene and screening of over several hundred individual clones are required to obtain cells with high gene copy numbers [4]. Consequently, the process for DHFR-mediated gene amplification is a time-consuming and laborious step for cell line construction. Here, we present a novel concept to accelerate gene amplification through cell cycle checkpoint engineering [5]. In our knowledge, there is no previous report which focused on controlling cell cycle checkpoint to enhance the efficiency of DHFR gene amplification system.

## Materials and methods

A small interfering RNA (siRNA) expression vector against Ataxia-Telangiectasia and Rad3-Related (ATR), a cell cycle checkpoint kinase, was transfected into Chinese hamster ovary (CHO) cells. The effects of ATR down-regulation on gene amplification and productivity in CHO cells producing green fluorescent protein (GFP) and monoclonal antibody (mAb) were investigated.

## Results and discussion

### Analysis of GFP expression level during gene amplification process

The ratio of GFP-expressing cells was evaluated by flow cytometry analysis during the gene amplification process at 100-, 250-, and 500-nM MTX concentrations. In the process of gene amplification at all MTX concentrations, the pools of ATR-downregulated cells showed a much higher percentage of GFP-positive cells as compared with the pools of mock cells. At 100-nM MTX concentration, the percentage of GFP-positive cells in the CHO-siATR cell pool was 18.7 % of total cells, which was approximately twice of the 8.4 % in the mock cells. At 250- and 500-nM MTX concentrations, CHO-siATR cell pools had 28.6 and 39.2 % GFP-positive cells, respectively, which were up to six times higher than the 4.6 and 6.8 % of the pools of mock cells.

### Comparison of IgG productivity

IgG-producing cell lines were generated to confirm the previous results obtained in GFP-producing cell lines. The ATR-downregulated cells showed a significant increase in specific production rate of an average of 0.08 pg cell^−1 ^day^−1^, which was approximately four times higher than the average of 0.02 pg cell^−1 ^day^−1 ^in the mock cells. The volumetric productivity of each cell line was also investigated to evaluate the influence of ATR downregulation. The volumetric productivity of ATR knockdown cells was an average of 0.035 mg L^−1 ^day^−1^, which was approximately three times higher than the average of 0.013 mg L^−1 ^day^−1 ^of the mock cells, suggesting that ATR knockdown generated the pool of higher-producing cells during the gene amplification process.

### Estimation of amplified transgene copy number

Quantitative real-time PCR was used to estimate the amplified transgene copy number of GFP-producing cell lines during the gene amplification process. The average copy number of ATR-downregulated cells was 15.4 ± 0.8, 27.6 ± 0.3, and 62.0 ± 2.9 at 100-, 250-, and 500-nM MTX concentrations, respectively. These numbers were up to 24 times higher than 3.98 ± 0.09, 2.20 ± 0.03, and 2.59 ± 0.07 of the mock cells. Interestingly, the amplified transgene copy numbers in the pools of ATR-downregulated cells were increased proportionally with the MTX concentration. The amplified transgene copy numbers in the IgG-producing cells were also investigated during the gene amplification process at 100-nM MTX concentration. The amplified light- and heavy-chain copy numbers of the pool of ATR knockdown cells were 13.2 ± 3.8 and 11.8 ± 1.8, respectively, which were up to seven times higher than 6.95 ± 0.07 and 1.68 ± 0.04 of the mock cells. The results from both the GFP- and IgG-producing cells showed that the pools of ATR-downregulated cells had much higher amplified transgene copy numbers as compared with the pools of mock cells during the gene amplification process.

## Conclusions

In conclusion, we have demonstrated that gene amplification can be accelerated by the downregulation of a cell cycle checkpoint kinase, ATR, and a pool of high-producing cells can be rapidly derived in a short time after MTX treatment. This novel method focuses on generating more high-producing cells in a heterogeneous pool as compared with the conventional method and would thus contribute to reducing the time and labor required for cell line establishment by increasing the possibility of selecting high-producing clones.
